# Recent Advances in Biocompatible Ionic Liquids in Drug Formulation and Delivery

**DOI:** 10.3390/pharmaceutics15041179

**Published:** 2023-04-07

**Authors:** Rahman Md Moshikur, Rebecca L. Carrier, Muhammad Moniruzzaman, Masahiro Goto

**Affiliations:** 1 Department of Chemical Engineering, College of Engineering, Northeastern University, 360 Huntington Avenue, Boston, MA 02115, USA; 2 Department of Chemical Engineering, Universiti Teknologi PETRONAS, Seri Iskandar 32610, Perak, Malaysia; 3 Department of Applied Chemistry, Graduate School of Engineering, Advanced Transdermal Drug Delivery System Center, Kyushu University, 744 Motooka, Nishi-ku, Fukuoka 819-0395, Japan

**Keywords:** biocompatible ionic liquid, drug solubility, drug formulation, drug delivery, bioavailability

## Abstract

The development of effective drug formulations and delivery systems for newly developed or marketed drug molecules remains a significant challenge. These drugs can exhibit polymorphic conversion, poor bioavailability, and systemic toxicity, and can be difficult to formulate with traditional organic solvents due to acute toxicity. Ionic liquids (ILs) are recognized as solvents that can improve the pharmacokinetic and pharmacodynamic properties of drugs. ILs can address the operational/functional challenges associated with traditional organic solvents. However, many ILs are non-biodegradable and inherently toxic, which is the most significant challenge in developing IL-based drug formulations and delivery systems. Biocompatible ILs comprising biocompatible cations and anions mainly derived from bio-renewable sources are considered a green alternative to both conventional ILs and organic/inorganic solvents. This review covers the technologies and strategies developed to design biocompatible ILs, focusing on the design of biocompatible IL-based drug formulations and delivery systems, and discusses the advantages of these ILs in pharmaceutical and biomedical applications. Furthermore, this review will provide guidance on transitioning to biocompatible ILs rather than commonly used toxic ILs and organic solvents in fields ranging from chemical synthesis to pharmaceutics.

## 1. Introduction

The pharmaceutical industry faces significant challenges when developing new drugs. In particular, many drugs exhibit poor bioavailability, which is attributed to their limited solubility in physiological fluids/water, poor permeability and inadequate absorption in the gastrointestinal tract, rapid metabolism, and/or degradation during systemic circulation [1,2]. Several organic solvents, including acetone, ethanol, isopropyl alcohol, dimethyl sulfoxide, dimethylformamide, and pyridine, are commonly used to dissolve these drug molecules. However, organic solvents present in pharmaceutical products are not accepted by regulatory authorities because they have severe adverse effects on the human body, including acute toxicity and carcinogenicity [2,3]. Various formulation techniques, including salt or prodrug formation, solid dispersions, nanoparticles, nanoemulsions, crystal engineering, hydrate and solvate preparations, and micellar systems, have been employed to design effective formulations of poorly soluble drugs [1,2]. Green techniques are attractive for the efficient formulation and delivery of poorly soluble medicines with negligible undesirable systemic effects.

Ionic liquids (ILs) have emerged as alternatives to organic solvents. Generally, ILs are molten organic salts of unsymmetrical organic cations and inorganic or organic anions with a melting point at or below 100 °C [4,5,6]. ILs have been extensively used to alter the physicochemical and biopharmaceutical properties of drug molecules to improve solubility, skin and intestinal permeability, formulation stability, and pharmacokinetic and pharmacodynamic properties [1,2]. Currently, three generations of ILs are available for various applications depending on their chemical structure and properties. First-generation ILs, specifically 1-butyl-3-methylimidazolium tetrafluoroborate and 1-butyl-3-methylimidazolium hexafluorophosphate, have unique physical and thermal properties, such as a low melting point, high thermal stability, low vapor pressure, and a wide range of fluidity [7]. These ILs are composed of dialkyl-imidazolium or alkyl-pyridinium cations with weakly coordinating anions, such as tetrafluoroborate, hexafluorophosphate, bis(trifluoromethylsulfonyl)amide, and methyl sulfate. The hexafluorophosphate salts of butyl, hexyl, and octyl-3-methylimidazolium cations have been explored as drug reservoirs for the prolonged controlled release of sucrose and dexamethasone [8]. Although first-generation ILs have attracted attention for their unique physical properties, they are sensitive to water and air and have poor biodegradability and aquatic toxicity [9]. Second-generation ILs are air- and water-stable, with tunable physical and chemical properties that allow them to be applied in lubricants, metal ion complexes, and energetic materials [1,10]. These ILs comprise biocompatible cations and anions derived from natural, renewable sources, including carbohydrates and amino acids. The third generation of ILs are prepared using mostly biocompatible and natural ions, such as choline, amino acids, and fatty acids, which have well-known biological activities [1,10,11]. Third-generation ILs comprising biologically active ions are particularly suitable for biopharmaceutical applications. Compared with first- and second-generations ILs, these ILs offer various unique advantages, such as low manufacturing costs, simplicity in synthesis, controlled polymorphism, and eco-friendly properties [10,11]. Biocompatible ILs are being increasingly exploited in pharmaceuticals because of the advantages they offer in terms of the solubility, formulation stability, and effective delivery (e.g., via oral, topical, and transdermal routes) of drugs, compared with conventional ILs. Recently, deep eutectic solvents (DES) have several advantages over traditional ILs such as their ease of preparation (by simply mixing the components with gentle heating) and easy availability from relatively inexpensive components [12,13]. In addition, no future purification is required, in contrast to ILs. Although ILs and DESs have a lot in common characteristics, specifically when it comes to physical properties and applications, from a chemical point of view, these are two separate groups of substances [12,13].

**Figure 1:**
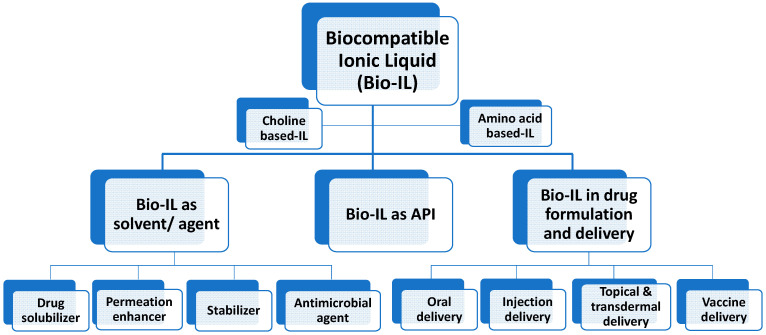
Applications of Bio-IL in drug formulation and delivery systems.

The aim of this review is to provide an integrative vision for applying biocompatible ILs in drug formulations and delivery systems. This review specifically discusses the technologies and strategies developed to control the biopharmaceutical properties of ILs and describes the state-of-the-art research on IL biological activity and biomedical applications. In addition, this review will explain the application of biocompatible ILs for continuous pharma manufacturing and provides future perspectives towards improving their performance in pharmacology. The intension of this review is to provide an update on and overview of the potential strategies used in the development of IL-based drug formulations and delivery systems within the last five years. An overall summary of the topics considered for this review is shown in Figure 1.

## 2. Biocompatible Ionic Liquids (Bio-ILs) in Drug Formulation

ILs are widely applied to pharmaceutics and medicines, which inevitably results in their direct contact with the living body, requiring an assurance of their long-term biocompatibility and safety in the patient’s body [1,2]. The ideal approach is to design bio-ILs using IL-forming cationic and anionic moieties derived from biocompatible materials, such as choline derivatives, amino acids, fatty acids, carboxylic acids, and non-nutritive sweeteners [14]. The environmental and economic issues of conventional ILs and commonly used organic solvents, such as toxicity, lack of biodegradability, and high prices, can be addressed using these bio-renewable and natural compounds to prepare bio-ILs [3,14]. Recently, task-specific chiral ILs (by introducing chiral centers either in the cations or anions) have been developed for sustainable pharmaceutical and food applications because of their abundance, nontoxicity, biodegradability, biocompatibility, relatively low price, and environmentally friendly behavior [15,16,17]. The perseverance of the scientific community has led to the wide use of several types of bio-ILs in drug formulations and delivery systems. Among these bio-ILs, choline and amino acid-based bio-ILs have been used significantly due to their numerous advantages.

### 2.1. Choline-Based Bio-ILs

**Figure 2:**
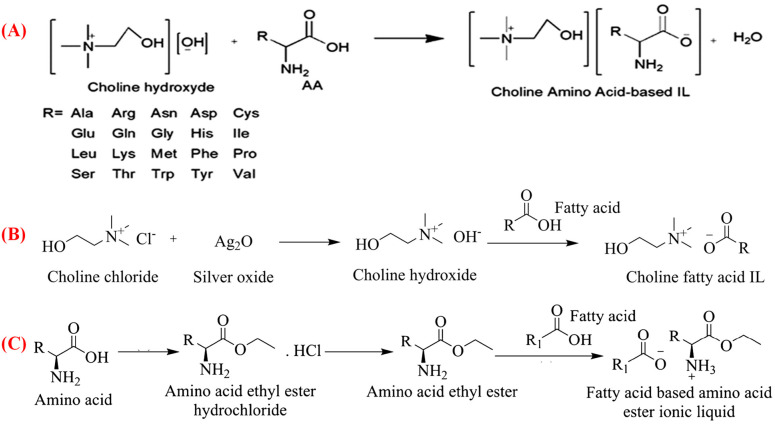
General synthesis procedures for (**A**) choline amino acids bio-ILs [20], (**B**) choline fatty acids bio-ILs [26], (**C**) IL-forming amino acid ester cation and amino acid fatty acid bio-ILs [27]; reproduced with permission from refs.

Numerous studies have shown that choline is a promising cation for preparing bio-ILs, owing to its intrinsic biodegradability and lower toxicity relative to other cationic moieties, including ammonium, phosphonium, imidazolium, and pyramidion [14,18]. The National Academy of Sciences and the United States Food and Drug Administration (FDA) have added choline to the human vitamin list and “generally regarded as safe” (GRAS) list. Choline is a precursor of the neurotransmitter acetylcholine and is an integral part of cell membrane-abundant phospholipids, namely, phosphatidylcholine and sphingomyelin [19]. Choline-based ILs are mainly synthesized using the salt of choline halides, such as choline chloride and choline iodide, as the IL-forming cation source [14]. Choline hydroxide is one of the prominent ILs synthesized from the metathesis of choline chloride with a metal oxide (silver oxide) or an anion exchange resin in the hydroxide form. A straightforward procedure is used to prepare several choline-based ILs, specifically, a neutralization reaction between choline hydroxide or choline bicarbonate solution (both commercially available) and slightly more than an equimolar amount of the desired acid, including amino acids, fatty acids, and carboxylic acids (Figure 2A,B) [14,20]. The procedures commonly followed to synthesize choline based-ILs include mixing these components in organic solvents for 12–24 h at room temperature or a specific temperature (i.e., 40 °C), followed by filtration to precipitate the excess acids and drying under high vacuum pressure to evaporate the aqueous organic solution [20]. Green and cost-effective choline-containing bio-ILs are synthesized using several natural and renewable materials, forming biodegradable bio-ILs with low toxicity and excellent physicochemical and biopharmaceutical properties [14,18]. Foulet et al. developed a series of choline-containing amino acids as bio-ILs (i.e., choline-glycine, -serine, -proline, -alanine, -histidine and -valine) and evaluated their toxicities and antimicrobial activities [21]. In another study, Raihan et al. prepared choline-containing glycine, alanine, proline, serine, leucine, isoleucine, and phenylalanine to investigate their cytotoxicity and drug solubilization efficiency [22]. Tenner et al. synthesized a series of choline-organic acid-based bio-ILs (i.e., choline-germanic acid, citronellic acid, octanoic acid, decanoic acid, hexenoic acid, salicylic acid, and glutaric acid) to enhance the transdermal delivery of several small and large molecules [23]. Several choline-containing fatty acids and *N*-lauroyl-amino acids have been developed recently as promising green alternatives to traditional surfactants in biomedical applications [24]. Choline-containing bio-IL buffers, namely Good’s buffers, have been prepared with different alkylamino methanesulfonate anions, buffering within pH 6 to 8 and offering high aqueous solubility, precipitation suppression during biochemical reactions, and stability against enzymatic and non-enzymatic degradation, compared with the use of more common buffers, such as phosphate, tris(hydroxymethyl)aminomethane, and borate [25]. Pedro et al. used choline-based bio-ILs to prepare self-buffering Good’s buffers as alternative preservation media to maintain the integrity and stability of recombinant small RNAs [25].

### 2.2. Amino Acid-Based Bio-ILs

Amino acids, one of the cheapest and most abundant biomaterials, can be easily converted into both IL-forming anions and cations for synthesizing bio-ILs. Using amino acids offers a sustainable route to ILs with low toxicity and high biodegradability—essential features of green ILs [1,14]. Amino acid-based ILs are prepared by converting cationic moieties into hydroxides using an anion exchange resin and then neutralizing these hydroxides with an equimolar amount of amino acids as IL-forming anions [14,28]. Several IL-forming cations, such as choline, ammonium, imidazolium, and phosphonium, have been used to synthesize amino acid-containing ILs with favorable physicochemical and thermal properties [1,14]. Amino acids are also used as cationic moieties with different strong acid-derived anions (nitrate, chloride, perchlorate, and trifluoromethane sulfonate) to synthesize bio-protic ILs [28]. Recently, Furukawa et al. converted the proline amino acid, a potent cation, with minimum cytotoxicity to formulate an IL active pharmaceutical ingredient (API) [29]. Moshikur et al. successfully synthesized amino acid esters to serve as biocompatible cationic moieties from a series of amino acids (Figure 2C). They converted several fatty acids into oil-miscible hydrophobic green ILs, envisaging their biomedical applications [27,30]. Shimul et al. used cationic amino acid ester to prepare bioactive phenolic ILs as green substitutes of typical toxic solvents for solubilizing poorly soluble bioactive natural preservatives [31]. Additionally, IL-forming moieties of protein-derived amino acids have attracted significant attention as bio-renewable compounds for synthesizing bio-ILs [14]. Glycine betaine is another biocompatible IL-forming cation that is considered a green alternative to choline for synthesizing bio-ILs. It has been combined with other compounds, and the system can be optimized by changing the alkyl side chain length and the nature of the anions to form DESs for biopolymer dissolution and processing [32].

## 3. Bio-ILs as Solvents/Agents

### 3.1. Drug Solubilizers

**Figure 3:**
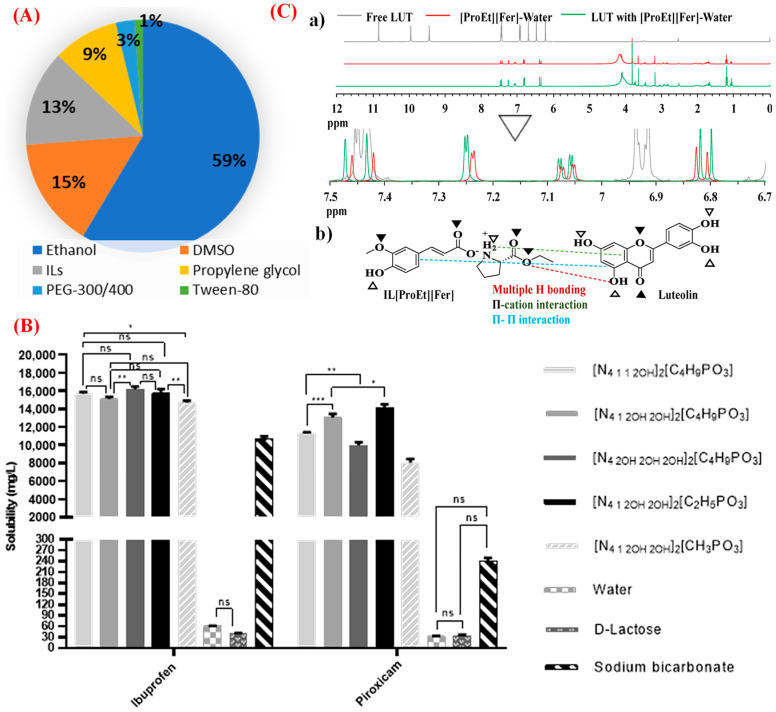
(**A**) A comparison of the use of ILs with commonly used pharmaceutical solvents/co-solvents and surfactants/co-surfactants [39]; (**B**) solubility of ibuprofen and piroxicam with ILs—commonly used excipients and water. Statistical significant differences are represented in asterisks: *** *p* < 0.0001, Ibuprofen: ** *p* = 0.0013 or *p* = 0.0085 and * *p* = 0.0119; Piroxicam: *** *p* = 0.0005, ** *p* = 0.0032 and * *p* = 0.0102, and ns—non-significant [34]; (**C**) Overlapped proton NMR spectra of IL-luteolin complexes (**a**); and the schematic diagram of the interactions between luteolin (Lut) and proline ethyl ester ferulate (IL[ProEt][Fer]) (**b**) [31], reproduced with permission from refs.

Dissolving a drug molecule in water or pharmaceutically accepted solvents is crucial in developing an effective drug formulation because it greatly affects drug pharmacokinetics and pharmacodynamics [1,2]. The challenges associated with poor solubility in water and biological media have continuously grown. However, many pharmaceutical strategies have been devised to improve the formulation and delivery of these drug molecules. Organic solubilizers, such as ethanol, methanol, acetone, and dimethyl sulfoxide, are commonly used to formulate drug molecules with limited aqueous solubility (Figure 3A). The residual presence of such organic solvents or co-solvents in a pharmaceutical product is not allowed by regulatory authorities because of the acute toxicity of the solvents; thus, there is a need for alternative solvents. As green and designable solvents, ILs have been successfully used to address the issue of poor water solubility for significantly improved druggability and bioavailability of drug molecules [2,33]. ILs have been used as suitable solvents, co-solvents, anti-solvents, hydrotropes, copolymers, and emulsifiers for many drug molecules [2]. ILs can significantly improve the physicochemical and biopharmaceutical properties of drugs by dissolving or transforming the drug molecules into the IL form. Many studies on IL drug solubilization have focused on quantifying the solubility of small molecule drugs and macromolecule therapeutics in neat and aqueous IL formulations (Table 1, entries 1–12). These investigations mainly use ILs of imidazolium, phosphonium, quaternary ammonium, and pyrrolidinium [2]. The use of ILs significantly improves the solubility of drugs compared with the use of aqueous solutions. The solubilities of ibuprofen and piroxicam in dianionic ILs are up to 300- and 480-fold higher than in water, respectively (Figure 3B) [34]. Recently, several bio-ILs, such as choline-containing amino acids, carboxylic acids, and fatty acids, have been used to improve drug solubility [1,2]. For example, the solubility of nobiletin in choline geranic acid (CAGE) is 450-fold higher than that in water [35]. Paclitaxel is solubilized and stabilized by choline amino acid ILs through steric and ionic effects, preventing drug aggregation over three months [22]. The solubility of paclitaxel in choline glycinate is 5585-fold higher than that in water—a significant improvement. Another study showed that the solubility of acyclovir is 581-fold higher than that in a mixture of water and ethanol—a commonly used organic solvent [36]. The solubility of drugs in ILs has been predicted using software, specifically conductor-like screening model for real solvents (COSMO-RS). Shimul et al. used COSMO-RS to predict the solubility of a bioactive compound, luteolin, in 180 ILs by combining 20 amino acid ethyl ester cations and 9 phenolic acid anions [31]. Lutfi et al. predicted the solubility of acyclovir in various ILs. They experimentally validated the predicted results, showing that the solubility of acyclovir in di- and tri-ethyl ammonium acetate-based systems is higher than that in the other systems investigated [37]. However, the solubilization mechanisms in neat ILs or aqueous IL formulations still need to be fully understood. Generally, drug dissolution depends on the formation of hydrogen bonds within IL-forming anions and water molecules, resulting in IL network stabilization in the aqueous phase [38]. In-depth experimental and molecular dynamics modeling studies have been conducted to explore the solubilization mechanism in ILs. Coutinho et al. used molecular dynamics simulations to elucidate the solubilization mechanism of the sparingly soluble cardiovascular drug, LASSBio-294, in IL aqueous solutions, demonstrating that multiple hydrogen bonding, π–π stacking, van der Waals interactions, and Coulombic contributions are vital for drug solubilization by ILs. Recently, nuclear magnetic resonance (NMR) has been used to experimentally determine the reason for the high solubility of drug molecules in ILs, demonstrating that multiple hydrogen bonding interactions between the drug and IL are the driving force behind drug solubilization by ILs (Figure 3C) [1,31]. Overall, a wide range of ILs have been used to improve the aqueous solubility of sparingly soluble drugs by forming strong interactions with drug molecules. Most of the work described here focuses on formulating and characterizing the physicochemical properties of ILs, with little work being performed to determine the in vitro toxicity. Thus, comprehensive research is still needed to determine whether the presence of ILs in formulations will affect in vivo drug delivery and whether the IL–drug interactions that lead to improved solubility will alter the therapeutic efficacy. In addition, a deep understanding of the structural interactions of IL–drug complexes in the presence of water is necessary for developing IL-mediated drug formulations because the formulations ultimately come into contact with water in biomedical applications.

### 3.2. Permeation Enhancers

**Figure 4:**
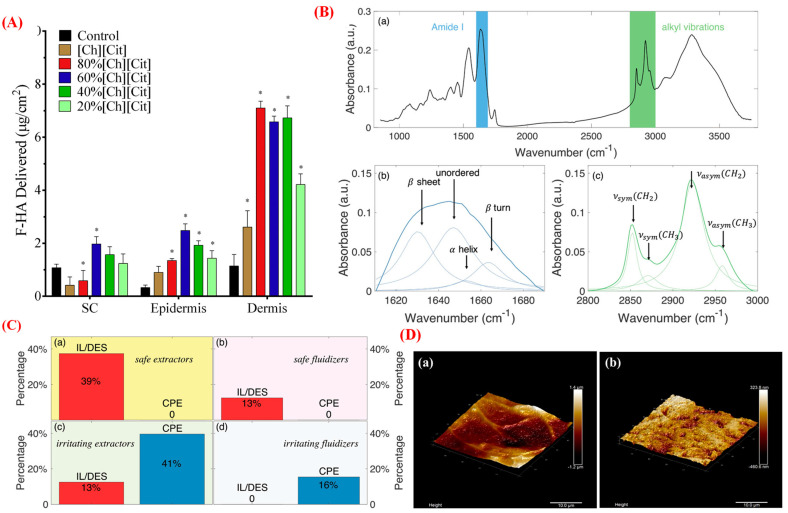
(**A**) The total amount of fluorescence hyaluronic acid (F-HA) determined in SC (stratum corneum), epidermis and dermis layer of the skin using choline citrate. Data represented as mean ± SD for *n* = 3 and * p < 0.05 compared with the control group [45]; (**B**) FTIR spectra of blank SC samples (**a**) with deconvoluted peaks in the amide I region (**b**) and lipid region (**c**) [46]; (**C**) A comparison of percentages of ILs/DESs (red) and CPEs (blue), representing (**a**) safe extractors, (**b**) safe fluidizers, (**c**) irritating extractors, and (**d**) irritating fluidizers [46]; (**D**) AFM images of SC samples treated with PBS solution (**a**) or IL-ME (**b**) [48]; reproduced with permission from refs.

The skin permeability of drug molecules remains a significant challenge due to the presence of a formidable barrier, namely, the skin’s stratum corneum (SC). The SC is the skin’s outermost layer and comprises corneocytes tightly packed in a lipid matrix [40]. Several technologies have been developed to transport small and macromolecular drugs across the skin, including ultrasound, iontophoresis, microneedle, and chemical permeation enhancers (CPEs). CPEs can penetrate the skin barrier by altering the lipid structure or disrupting the SC [41]. However, only a few of these CPEs are used in pharmaceutical applications because most CPEs lead to acute skin irritation or toxicity [2,40]. Recently, studies on ILs have been focused on exploring green alternatives to conventional organic solvents and materials/agents for the transdermal drug delivery of drug molecules [40]. Most of the research on the use of ILs aims to enhance the skin penetration of small and large molecules, including acyclovir, methotrexate, dantrolene sodium, rifampicin, etodolac, 5-fluorouracil, fluconazole, salicylic acid, caffeine, amphotericin, and peptides and proteins [2,40]. The permeation profiles of mannitol, cefadroxil, and diltiazem in the presence of other ILs (including phosphonium, morpholinium, pyrrolidinium, and 1,4-diazabicyclo [2.2.2]octane-based salts) have also been determined, highlighting various properties of ILs as solubilizing, enhancing, and irritating agents [42,43]. However, the inherent toxicity of some ILs necessitates the exploration of biocompatible ILs, motivating the design of next-generation ILs. Several bio-ILs, such as choline-based fatty acids and organic acids, amino acid ester-based fatty acids, and phosphatidylcholine-based fatty acids, have been used as promising skin permeation-enhancing agents to assist the topical/transdermal delivery of drug molecules [2,40]. Hattori et al. have used a CAGE IL as a solubilizing and skin permeation-enhancing agent to enhance the transdermal absorption of nobiletin, which resulted in 20-times more bioavailability than oral administration of the parent drug [35]. Bekdemir et al. have used a similar CAGE IL for the transdermal diffusion of thrombin-sensitive nanosensors into the skin dermis [44]. Several biocompatible hydrophobic fatty acid-based amino acid ILs have been investigated as solubilizing and skin permeation-enhancing agents for ibuprofen and a peptide drug, and have demonstrated a higher degree of drug permeation compared with the conventional CPE Transcutol [27]. Choline containing carboxylate ILs have been used to improve the dermal delivery of hyaluronic acid (Figure 4A) [45].

The mechanisms by which ILs or API-ILs exhibit improved skin permeability are not fully understood. They mainly depend on the unique structural and physicochemical characteristics of the IL. ILs can extract or fluidize lipids from SC bilayers and enhance the penetration of drug molecules via different pathways, including the intracellular, intercellular, and follicular routes (Figure 4B) [2,40]. Generally, hydrophilic ILs fluidize the lipid layers from the SC by disrupting the tight packing of phospholipid bilayers. In addition, hydrophobic ILs facilitate partitioning by weakening the inter-lipid interactions in the epithelial membrane of the SC. Attenuated-total-reflectance Fourier transform infrared (ATR-FTIR) has been used to investigate the influence of neat ILs/DESs on the skin’s structure (Figure 4C) [46]. The reduction of the peak area between 2800 cm^−1^ and 3000 cm^−1^ in the ATR-FTIR spectra indicates the extraction of lipids from the SC due to the symmetric and asymmetric stretching vibrations of the alkyl groups, while the shifting of these peaks indicates the fluidization of lipids from SC bilayers. Furthermore, the characteristic absorption bands of amide I (≈1650 cm^−1^) and amide II (≈1550 cm^−1^) reveal information regarding the keratin structure of proteins in the horny layer of the skin. Differential scanning calorimetry has been used to investigate structural changes in the SC. In particular, if there is an endothermic peak in the thermogram, attributed to the melting of lipid bilayers, the peak shifts with increased skin permeation [47]. Atomic force microscopy (AFM) examination can reveal the surface topography of SC samples to explore the effects of IL on the SC structure (Figure 4D) [48]. Confocal laser scanning microscopy has been used to visualize the effect of ILs on drug permeation across the skin via the intercellular lipid pathway [47]. Molecular dynamics simulations have been conducted to investigate the interaction of a model cell membrane with an IL, revealing the insertion of cationic headgroups of ILs into the cell membrane [49].

### 3.3. Macromolecular Therapeutic Stabilizers

**Figure 5:**
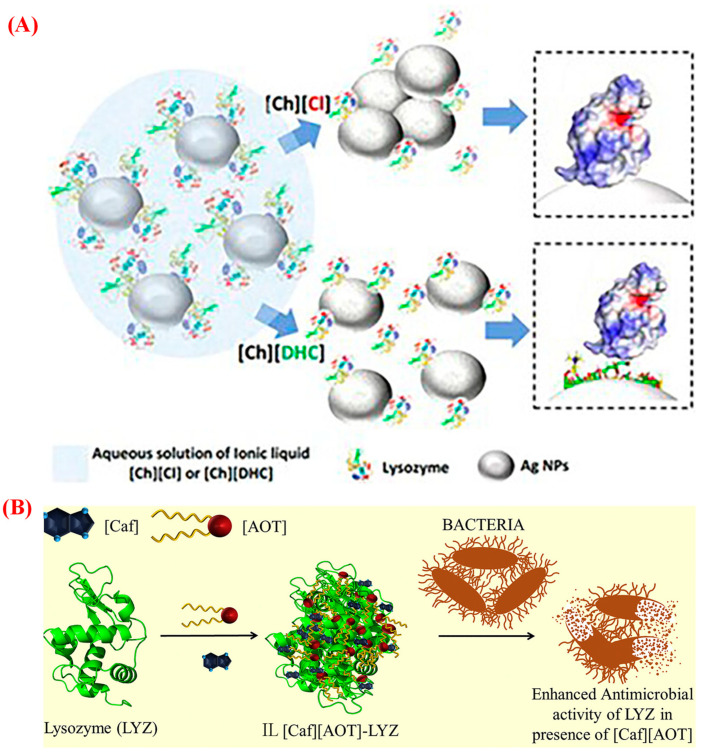
(**A**) Schematic representation of the impact of aqueous choline dihydrogen citrate (IL[Ch][DHC]) and choline chloride (IL[Ch][Cl]) on the protein–silver nanoparticle conjugates. IL[Ch][Cl] containing protein is adsorbed on the surface of nanoparticles, in contrast, the dihydrogen citrate anions of IL[Ch][DHC] interact with the silver surface with the oxygen atom (shown in the square box) [54]. (**B**) the enhancement of the antimicrobial activity of the lysozyme (LYZ) against Gram-positive and Gram-negative bacteria [56], reproduced with permission from refs.

ILs have emerged as potential solvents in biotechnology, especially as stabilizers of proteins, nucleic acids, and enzymes [50]. They play a significant role in improving the stability and preventing the unfolding or aggregation of many biopharmaceuticals. The use of ILs can extend the protein shelf life and address the formulation challenges of these therapeutics in aqueous buffered solutions [50,51]. Imidazolium-based ILs are widely used to gain mechanistic insights into the complex interactions of ILs with macromolecules. Ammonium-based ILs also exert low toxicity and offer thermal and conformational stabilization [50,52]. Recently, bio-ILs have become one of the most promising candidates for stabilizing biopharmaceuticals due to their natural sources and noticeable effect on protein stability. Shmool et al. developed a choline chloride bio-IL-based strategy to hinder stress-induced protein conformational changes and predict the protein aggregation propensity and thermodynamic equilibrium of the fresh immunoglobin G4 antibody in water and various IL solutions [53]. The protein aggregation propensity reduces with increasing IL concentrations over an extended 365 days of storage, even under stress conditions. The same choline chloride IL was used to determine the mechanism by which lysozyme–nanoparticle interactions are stabilized, and this system was compared with other choline dihydrogen citrate bio-IL-based systems to investigate the role of anions in the ILs (Figure 5A) [54]. Bisht et al. used a series of choline-based bio-ILs to investigate the stability of the chymotrypsin structure against thermal denaturation and revealed that choline acetate IL-containing chymotrypsin shows a high stabilizing capacity among all studied ILs and choline hydroxide solution [55]. The presence of ILs in lysozyme formulations not only maintains the necessary hydrophobicity of the active site of the enzyme but also helps with bacterial cell wall adsorption through lipid-like activity (Figure 5B) [56].

Many researchers have successfully explored and revealed the potential of ILs as activity enhancers as well as macromolecule stabilizers [50,51]. Although there is still much to learn about how proteins interact with ILs, the mechanism by which macromolecules in ILs are stabilized or destabilized has been investigated using experimental data and molecular dynamics simulations, revealing the critical role of interactions among ILs, macromolecules, and water [50,51]. ILs have emerged as potent solvents to solubilize or stabilize macromolecules in neat ILs or aqueous IL solutions owing to their favorable physicochemical properties, such as hydrophobicity, ion tunability, and hydrogen bonding ability, and their biopharmaceutical properties. Molecular dynamics simulations have demonstrated that the IL anion significantly affects protein stability at high IL concentrations by interacting with positively charged residues and promoting the refolding of proteins [50]. These interactions aid in forming hydrogen bonds and restructuring secondary structural elements, resulting in the protein’s increased thermal stability.

### 3.4. Antimicrobial Agents

The inappropriate and excessive use of antibiotics has led to the emergence of multidrug-resistant pathogens, which may kill more than 10 million people annually by 2050, accounting for 45% of all deaths [57]. ILs have excellent antimicrobial properties, opening new avenues to address the challenges posed by antibiotic-resistant pathogens [1,57]. ILs can interact with microbes by crossing cell wall membranes and altering the characteristics of cell wall membranes, including membrane fluidity, viscoelasticity, and phospholipid composition [57]. Generally, ILs are attracted to the cell membrane of microbes through electrostatic forces between the positively charged components of ILs and the negatively charged regions of the microorganism’s cell membrane or wall. Hydrophobic interactions between the cationic side chains of ILs and cellular lipids allow the insertion of the IL molecules into the membrane, which ultimately disrupts and disintegrates the phospholipid bilayer with leakage of the intracellular cytoplasm [57]. Benedetto et al. investigated this mechanism using choline and imidazolium-based ILs. They demonstrated that both cations can permeate the lipid layers of bio-membranes by occupying 2–10% of the bilayer volume [58]. However, the antimicrobial action of ILs is related to the chemical structure of ILs, which is similar to that of well-known cationic surfactants, suggesting that ILs can aggregate in solution to form amphiphilic micelles, which disrupt the integrity of the microbial cell wall/membrane.

**Table 1:** Pharmaceutical applications of Bio-ILs as solvents, solubilizing and encapsulating agents, stabilizers and pharmaceuticals.

No.	Drug	ILs	Role of ILs	Main Findings	Ref.
1.	Hypoxia-inducible factor (HIF) as cancer drug	Pyridinium-based-ILs	Solvents	a. ILs inhibited the cancer cells viability compared to the normal cells. b. ILs suppressed the mitochondria and HIF-1α- dependent glucose metabolic pathway in hypoxic cancer cells.	[59]
2.	Diacerein	Betaine- and carnitine-based ILs	Solubilizing and stabilizing agent	a. Enhanced the solubility and stability of drug. b. Exhibited strong bactericidal activity against pathogens. c. IL-mediated formulation increased the ocular residence time through mucoadhesion.	[60]
3.	Lysozyme Bovine serum albumin	[Cho][H_2_PO_4_] [Cho][C_4_F_9_SO_3_] [C_2_MIN][C_4_F_9_SO_3_]	Solubilizing and encapsulating agent	a. Maintained the globular folded structure and hydrolytic activity of the proteins in the presence of ILs. b. Encapsulated LYZ by the ILs.	[61]
4.	Luteolin	[ProEt][Fer] [ProEt][Van] [ProEt][Cou] [ProEt][Ben]	Solubilizing agent	a. LUT’s solubility in ILs showed 880-fold higher than in water. b. ILs revealed as green solvents. c. Exhibited superior organoleptic properties on red apple slices.	[31]
5.	Luteolin	[Cho][Ole]	Encapsulating agent	a. Formed spherical micelles with a mean particle size of 73 nm. b. Exhibited enhanced aqueous solubility with excellent encapsulation efficiency (94.3%). c. Showed higher antibacterial activity with excellent food preservation activity than toxic chemical preservatives.	[62]
6.	Ibuprofen	[Cho]:[Van], [Gal], [Sal]	Solubilizing agent	Significantly increased the drug solubility compared with conventional hydrotropes.	[63]
7.	Piroxicam Ibuprofen	[N_4,1,2OH,2OH_]_2_ [C_2_H_5_PO_3_]	Solubilizing agent	a. Significantly increased the solubility up to 300-fold compared with water. b. Decreased lipophilicity of drugs in the presence of the ILs. c. No significant toxicity observed against fibroblast cells.	[34]
8.	Ibuprofen	Dianionic ILs	Solubilizing agent	a. Solubility increased up to 40-fold compared with water. b. Lipophilicity decreased in the presence of ILs.	[64]
9.	Diclofenac sodium	[Cho][Ace]	Hydrophilic agent in drug carrier	Exhibited excellent biodegradability, stimuli-responsive properties, and biocompatibility.	[65]
10.	Chlorambucil	[C_4_MIM][Tf_2_N]	Hydrophilic inner phase	a. Showed a burst release at first 4 h with excellent antitumor activity against breast cancer cells. b. Exhibited a reduced the toxicity in the zebrafish animal model.	[66]
11.	Photosensitizer	[C_12_MIM][Br]	Surfactant and drug encapsulating agent	a. High drug-loading and encapsulation efficiency compared with conventional surfactant-containing nanocarriers. b. Higher ability to produce cytotoxic singlet oxygen with a sustained release profile.	[67]
12.	Epirubicin	[P_6,6,6,14_][Cl]	Drug carrier	a. Exhibited good capability in delivering therapeutic to the sickness sites. b. Significantly decreased the adverse effects.	[68]
13.	DNA	lipid-like ILs	Drug carrier	a. Exhibited efficient gene transfection vectors with excellent biocompatibility. b. Formed cationic bilayers to deliver DNA into the targeted cell.	[69]
14.	Curcumin	[Cho][Ole]	Drug carrier	Significantly enhanced solubility and half-life of drug compared with free drug in water.	[70]
15.	Immunoglobin G4 (IgG4)	[Cho][Cl]	Protein stabilizer	a. Predicted the thermodynamic stability with aggregation propensity of protein IgG4. b. Aggregation propensity reduced with increasing IL concentration.	[53]
16.	Lysozyme (LYZ)	[Cho][Cl], [Cho][DHC]	Protein stabilizer	a. The aggregation behavior varied with ILs. b. ILs enhanced the stabilization of LYZ in the interfacial surface.	[54]
17.	Lysozyme	[C_2_MIM][Tf_2_N]	Nonpolar phase of the microemulsion	a. Enhanced the thermal stability of LYZ up to 120 °C. b. Enhanced the enzyme activity with the presence of IL.	[71]
18.	TCN Hydrogel sensor	Polyvinyl-imidazole	Polymeric network of the hydrogel	a. Exhibited excellent strain sensing sensitivity, short response time, high durability, and impressive temperature sensing sensitivity. b. Achieved rapid wound closure and condition monitoring.	[72]
19.	-	Choline-based bio-IL	Tissue scaffold	a. Formed in situ 3D printable bio-ionic ink hydrogel with ILs. b. Improved cellular adhesion and reduced potential fouling with excellent biocompatibility and cell proliferation.	[73]
20.	5-fluorouracil (5-FLU) Indomethacin (IND)	Sulfonate-ILs	crosslinking agents	a. Exhibited controlled and sustained drug release profiles. b. Stronger interactions of 5-FLU with beads, resulting a slower release than IND after 24h.	[74]
21.	calf thymus DNA	Noscapine-IL	Binding agent	Exhibited a strong and stable binding with the target DNA.	[75]
22.	Etodolac (ETO)	[ProEt][ETO]	Pharmaceuticals	a. Enhanced the solubility in the simulated nasal fluid owing to the ionization of drug. b. Improved the drug retention on the nasal mucosa with enhanced the nose-to-brain delivery. c. Suppressed the production of PGE2 in the intranasal administration.	[76]
23.	Ibuprofen	[AAOR][Ibu]	Pharmaceuticals	a. IBU-ILs does not affect the binding affinity of drug to BSA. b. ILs are practically not toxic. c. ILs effectively bound with BSA.	[77]
24.	Donepezil	dicarboxylic acid-based DPZ	Pharmaceuticals	Exhibited improved aqueous solubility and skin permeability compared to the free drug.	[78]
25.	Ketoprofen	Piperine-based IL	Pharmaceuticals	a. Exhibited enhanced aqueous solubility with excellent permeability across the rat skin. b. IL resulted in a 68% less paw swelling than mixture of free drug.	[79]
26.	Pilocarpine (Pilo)	Pilo- oligo-polyethylene glycol ILs	Pharmaceuticals	a. Exhibited a higher corneal permeability coefficient than the free drug. b. ILs does not show apparent toxicity to human corneal epithelial cells.	[80]
27.	lidocaine (Lid) procaine (Pro)	[Lid], [Pro]:[Sal]	Pharmaceuticals	Exhibited a higher aqueous solubility up to 10-fold compared to the free drug, indicating a potential drug delivery carrier for topical drug delivery.	[81]
28.	Salicylic acid	Betaine-, N-hexyl-nicotinamide-, benzalkonium-Sal	Pharmaceuticals	Exhibited higher antimicrobial activity with a lower cytotoxicity than free salicylic acid	[82]
29.	Salicylic acid	[AAOR][Sal]	Pharmaceuticals	a. Decreased the cytotoxicity toward various type of cells. b. Inhibited the production of the proinflammatory cytokine IL-6 in keratinocytes.	[83]
30.	Metformin (Met)	[Met]:[Ibu]	Pharmaceuticals	Exhibited excellent anti-diabetic and anti-inflammatory activities compared to the parent drugs.	[84]
31.	Lidocaine (Lid) imipramine (Imp) levamisole (Lev)	[Lid]:[Lau], [Ole], [Lin], [Ste] [Imp]:[Lau], [Ole], [Lin], [Ste] [Lev]:[Lau], [Ole], [Lin], [Ste]	Pharmaceuticals	a. Freely miscible in ethanol, N-methyl pyrrolidone, Tween 20, and isopropyl myristate. b. Oleate-based Lev formulation showed 2.6-and 5.4-fold higher in vitro and in vivo skin permeation capability, respectively, than the Lev salts.	[85]
32.	Methotrexate	[MTX]:[Cho], [ProEt], [AspEt], [TMA], [TBP]	Pharmaceuticals	a. Enhanced the aqueous solubility up to 5000-fold more than the free drug. b. ProEt-MTX exhibited 4.6-fold higher oral bioavailability compared to free MTX. c. Significantly reduced systemic toxicity compared to free MTX.	[86,87]
33.	Procaine (Pro)	[Pro]:[Sal], [Ibu], [Doc]	Pharmaceuticals	Significantly increased the aqueous solubility of Sal- and Ibu-based drugs but reduced with Doc anion.	[88]
34.	Favipiravir (Fav)	[Fav]:[Cho], [ProEt], [AlaEt]	Pharmaceuticals	a. Improved aqueous solubility by 78 to 125-fold compared to the free drug. b. Significantly increased the oral bioavailability compared to the free drug.	[89]
35.	Cinnarizine	cinnarizine decanoate	Pharmaceuticals	Significantly enhanced the apparent solubility in lipid solution compared to the free drug.	[90]

Abbreviations: [C_2_MIN], 1-ethyl-3-methylimidazolium; [C_4_MIM], 1-butyl-3-methylimidazolium; [C_12_MIM], 1-dodecyl-3-methyl imidazolium; [P_6,6,6,14_], tributyl(tetradecyl)phosphonium; [H_2_PO_4_]: dihydrogen phosphate; [C_4_F_9_SO_3_], perfluorobutylsulfonate; [Fer], ferulate; [Van], vanillate; [Pan], p-coumarate; [Ben], 4-hydroxybenzoate; [Gal], gallate; [Sal]’ salicylate; [Br], bromide; [Cl], chloride; [Ace], acetate; [DHC], dihydrogen citrate; [AAOR], amino acid alkyl esters.

## 4. Bio-ILs as Active Pharmaceutical Ingredients

**Figure 6:**
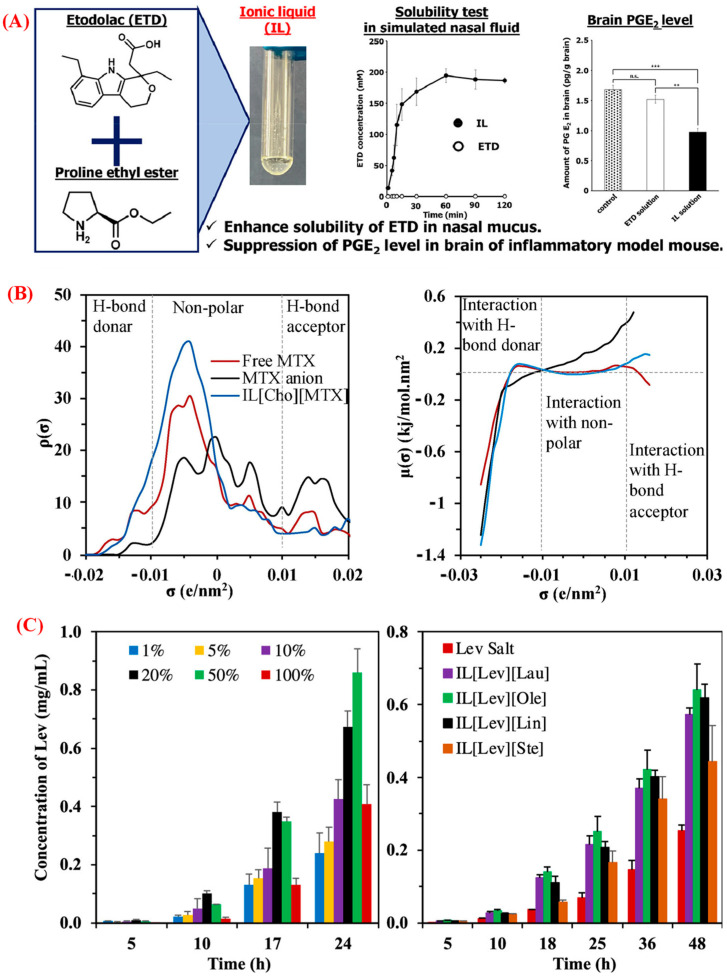
Schematic representation of the biopharmaceutical properties of (**A**) etodolac- containing API-ILs. Data represented as mean ± SD for *n* = 5 and *** *p* < 0.001, ** *p* < 0.01, n.s. *p* > 0.05 [76]; (**B**) the solute–solvent intermolecular interactions of free methotrexate (MTX), the MTX anion, and IL[Cho][MTX] in aqueous environments. The molecular surface polarity distributions (σ-profiles, **left**) and σ-potentials (**right**) represent the affinity of these compounds with water [86]; (**C**) effect of an oil phase (%) in the transdermal permeation of IL[Lev][Ole] (**left**) and the cumulative permeation profiles of the Lev salt in PBS and the Lev-ILs in oil (**right**) (Lev = levamisole; Lau = laurate; Ole = oleate; Lin = linoleate; Ste = stearate) [85], reproduced with permission from refs.

The API of a drug formulation is the main biologically active component developed to target a disease and produce a therapeutic effect. Unfortunately, 40–70% of marketed drugs fail to achieve their therapeutic efficacy because of their poor solubility, low bioavailability, and polymorphic conversion [2,91]. To address these issues, IL-forming counterions have been used to form IL-based APIs by transforming these drug molecules into ILs (Table 1, entries 22–35). This innovative API-IL technology enables the fine-tuning of the parent drug’s physicochemical and biopharmaceutical properties to increase solubility, thermal stability, and dissolution rate, as well as the suppression of polymorphism [1,2]. Generally, APIs can easily be protonated or deprotonated to form the salt form of the drug, depending on the difference in pKa values between the API and the other precursor compound. The difference in pKa value between the API and precursor should be >2 in order to successfully transfer a proton for conversion into an ionic salt [92]. API-ILs have been used in drug formulations to provide excellent pharmacokinetics and pharmacodynamics profiles and to enable different routes of administration, such as oral, injection, topical, and transdermal [93]. API-ILs are commonly prepared using the metathesis reaction, which employs a substantial amount of organic solvents, including methanol, ethanol, chloroform, acetone, isopropanol, and tetrahydrofuran, resulting in the formation of unwanted contaminants [2,93]. Recently, neutralization or mechanochemical synthesis has been used as a faster, solvent-free, and higher-yield method for API-IL preparation. An ion exchange resin is used to form an ionized hydroxyl ion from halide counterions, facilitating the neutralization reaction between the constituent IL ions [93]. An alternative route to API-ILs uses isolation-free manufacturing combined with co-processing via spray drying to take advantage of inaccessible API-IL forms [94]. Many cations and anions have been used to improve the pharmacological activity of a drug molecule. Currently, IL-forming counterions are chosen from compounds on the GRAS list of sections 201(s) and 409 of the Federal Food, Drug, and Cosmetic Act, which have been reviewed and approved by the US FDA. The selection of counterions is essential for the successful design of API-ILs because it is currently difficult to predict what ion combinations will result in ILs. The critical findings of studies on API-ILs indicate that certain IL-forming counterions can significantly enhance APIs in terms of solubility, release profile, permeability, thermal stability, toxicity, bioavailability, and drug efficacy [2,93]. The type of cations constituting ILs can significantly affect the physicochemical and biological characteristics of API-ILs. For example, the aqueous solubility of favipiravir in API-ILs containing choline and amino acid esters (AAEs) is at least ten times higher than that of the free drug, resulting in higher bioavailability [89]. Similarly, improved solubility in both water and simulated body fluids (SBF) can be obtained (at least 5000 times higher than that of the free drug) when the anticancer drug methotrexate is combined with choline, ammonium, imidazolium, and AAEs [87]. Salicylate-containing API-ILs with AAEs are miscible with any ratio of water, indicating excellent solubility enhancement of the slightly water-soluble salicylic acid [30]. The solubility of proline ethyl ester-bearing etodolac is significantly improved in SBF, by 200-fold, compared with that of the parent drug, due to the enhanced drug retention in the nasal mucosal surface (Figure 6A) [76]. Similarly, dicarboxylic acid-containing donepezil exhibits excellent solubility in phosphate buffer solution [78]. Choline curcumin IL exhibited enhanced solubility in SBF with excellent aqueous stability and anticancer activity. A theoretical simulation of methotrexate that contained API-ILs predicted the solute–solvent intermolecular interactions of IL-drugs in aqueous environments [86]. The greater molecular surface polarity distributions in the H-bond donor regions led to superior intermolecular interactions compared to that of the free drug (Figure 6B). Similarly, hydrogen-bond breaking and rebuilding facilitated the interaction of curcumin and water molecules [95]. API-ILs comprising fatty acids and lidocaine, imipramine, and levamisole show free miscibility with pharmaceutically acceptable solvents/agents (i.e., ethanol, Tween 20, *N*-methyl pyrrolidone, and isopropyl myristate), resulting in a practical and translatable transdermal drug delivery platform for hydrophilic drugs (Figure 6C) [85]. The skin permeability and biological activity of API-ILs rely on the nature of the IL-forming counterion [93]. The anticancer activity of MTX can be increased by adding AAEs as low-toxicity cations, whereas choline-containing ILs with MTX display an antitumor activity similar to that of the parent drug [87]. *N*-methyl-2-pyrrolidone containing ibuprofen IL exhibits enhanced skin penetration with lower cytotoxicity than choline ibuprofen [96]. Amino acid alkyl ester salicylates exhibit significantly decreased cytotoxicity toward NIH/3T3 murine embryo fibroblasts and human HaCaT keratinocytes compared to the free drug. These API-ILs inhibit the production of proinflammatory cytokine IL-6 in keratinocytes. They exhibit a binding affinity toward bovine serum albumin and a pharmacokinetic profile similar to that of the free drug [83]. Metforminium ibuprofenate has better anti-diabetic and anti-inflammatory properties than the parent compounds [84].

## 5. Drug Delivery Applications of Bio-ILs

### 5.1. Bio-ILs in Oral Formulation and Delivery

**Figure 7:**
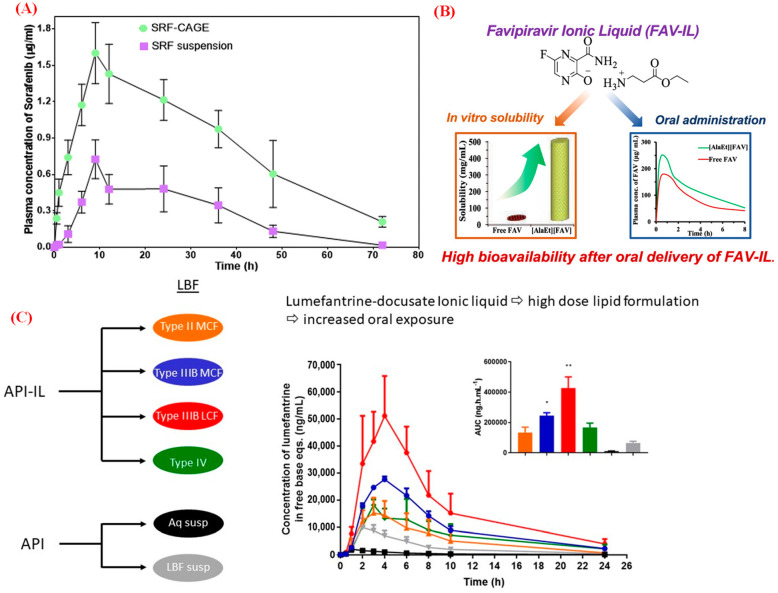
Schematic representation of the oral administration of (**A**) CAGE-containing sorafenib (SRF) drug [97]; and the IL forms of (**B**) favipiravir [89] and (**C**) lumefantrine [103]. Data are mean ± SD (*n* = 4). * *p* < 0.05) when compared to both suspensions. ** *p* < 0.05) from all other formulations; reproduced with permission from refs.

Oral administration has advantages over injection and other delivery methods, including ease of use, simple administration, high patient compliance, low production costs of oral formulations, and non-invasiveness [18,97]. However, some common challenges faced in the oral drug delivery route need to be considered, particularly poor solubility and permeability, high levels of P-glycoprotein efflux, pre-systemic metabolism, and high rates of drug molecule degradation [97]. To overcome these issues, IL-based formulations have been proposed as a unique strategy to solubilize and formulate problematic small and macromolecular drugs/therapeutics for the development of practical and translatable oral drug delivery systems (Table 2, entries 1–8) [18,98]. Many biological therapeutics, such as insulin, monoclonal antibodies, and immunoglobulin (IgG), have been successfully delivered via oral administration using ILs. Choline-based ILs (choline/glycolic acid molar ratio of 2:1, 1:1, and 1:2) can be prepared for the oral delivery of insulin and IgG. The IL-based formulation significantly enhances IgG penetration through intestinal mucus and epithelium [99]. Similarly, improved oral delivery of monoclonal antibodies into the intestinal mucosa can be achieved using choline and glycolate IL [100]. Choline germinate IL-containing formulations significantly enhance the paracellular transport of insulin by protecting it from enzymatic degradation and interactions with the mucus layer in the gastrointestinal tract [101]. These studies demonstrate the potential use of ILs for enhancing the oral delivery of macromolecular drugs. IL-based formulations are also considered potential alternatives for the oral delivery of small molecular hydrophobic drugs. For example, the choline oleate IL-based formulation significantly enhances the absorption of PTX delivered orally compared with the marketed chromophore EL-based formulation [102]. Similarly, the CAGE 1:2-containing formulation has been investigated for the oral delivery of hydrophobic drug sorafenib, resulting in an IL-based formulation with a peak plasma concentration, drug elimination half-life, and mean absorption time 2.2-, 2-, and 1.6-fold higher, respectively, than those of the parent drug suspension (Figure 7A) [97]. The bioavailabilities of proline ethyl ester-containing methotrexate and alanine ethyl ester-bearing favipiravir delivered orally are 4.6- and 1.9-fold higher than that of the respective parent drugs (Figure 7B) [86,89]. The solubility of lumefantrine docusate IL in lipid-based formulations is 80-fold higher than that of the free drug, resulting in improved plasma exposure (up to 35-fold higher) compared to the control lipid and aqueous suspension formulations of the free drug (Figure 7C) [103]. Although these IL-based oral delivery systems have successfully delivered both small and large therapeutic molecules, it is yet to be discovered if they are safe within the living body.

### 5.2. Bio-ILs in Injection Formulation and Delivery

The intravenous (IV) route or direct injection at the site of action offers a rapid onset of action and avoids first-pass metabolism. However, several challenges must be overcome in developing injectable formulations of poorly water-soluble drugs due to the high hydrophobicity and potential severe side effects of these drugs. Recently, IL-based formulations have been used to improve the biopharmaceutical properties of drugs delivered via injection (Table 2, entries 9–12). A choline germinate IL-based formulation of a chemotherapeutic drug (doxorubicin) has been developed for percutaneous injection into liver tumors in a rabbit liver tumor model, leading to consistent tumor ablation in the rabbit liver tumor model for prolonged periods [104]. An IL formulation of doxorubicin exhibits synergistic cytotoxicity against cultured HCC cells with a uniform drug distribution throughout the ablation zone when injected into liver tumors in the rabbit liver tumor model. Similarly, doxorubicin-loaded imidazolium IL ([C_4_MIN][PF_6_])–polydopamine nanocomposites combined with microwave irradiation have an apparent antitumor efficacy with high inhibition effects [105]. IL-mediated paclitaxel (PTX) shows excellent antitumor activity with a minor hypersensitivity effect in vitro compared to commercial cremophor EL-mediated paclitaxel (Taxol) [106]. An IL-based PTX formulation is similar to Taxol in terms of systemic circulation time and antitumor activity. CAGE IL-containing large molecular proteins, such as monoclonal antibodies, significantly enhance monoclonal antibody absorption by ≈200% after subcutaneous injections [107]. Taken together, IL-mediated injectable formulations open up new possibilities for developing effective and translatable drug delivery strategies for small molecule drugs and biological therapeutics.

### 5.3. Bio-ILs in Topical and Transdermal Delivery

**Figure 8:**
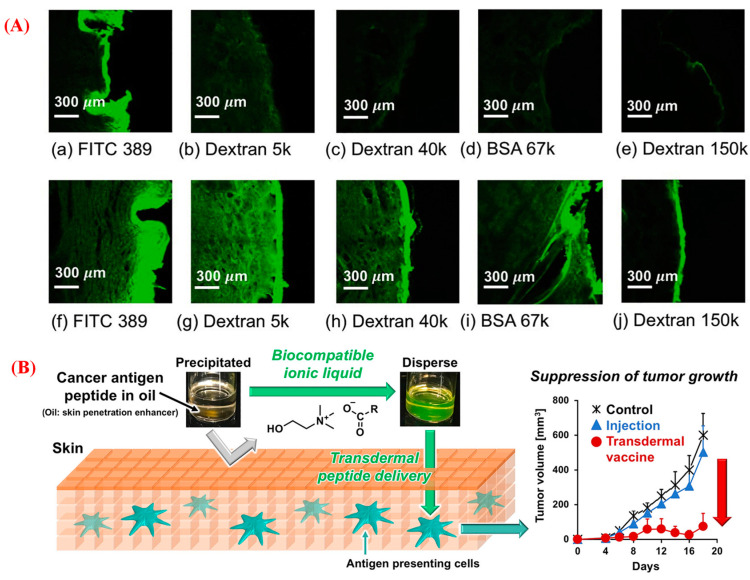
(**A**) Confocal microscopy showing the comparison of a variety of molecules solvated in PBS (**a**–**e**) and CAGE 1:2 (**f**–**j**) and transported through porcine skin. Fluorescence intensity was calibrated based on the stock solutions. FITC labelling was used except (**b**,**g**) which are TD [113], (**B**) IL-mediated micellar formulation enhancement of transdermal delivery of antigenic peptide using IL-in-oil microemulsion formulations [116]; reproduced with permission from refs.

Transdermal drug delivery has attracted attention as a non-parenteral administration technique due to its ease of application and termination, noninvasive nature, sustained therapeutic action, and better patient compliance [40,108]. In some circumstances, transdermal drug delivery can circumvent the first-pass metabolism of oral delivery and provide an acceptable therapeutic effect across the skin barrier. An oil-based formulation can facilitate the permeation of the drugs across the skin because it has excellent surfactant properties, and lipophilic oils act as skin penetration enhancers. After being applied to the skin, oils and their components are rapidly metabolized, and the resulting products are quickly excreted without any accumulation in the body, indicating they are potentially useful and safe penetration enhancers [109,110]. Recently, bio-ILs and DESs have been investigated in regard to their ability to increase skin permeability [18]. ILs have been used in different formulations, including microemulsions, nanoparticles, and (bio)polymer-based drug delivery systems (such as patches and membranes), to deliver poorly water-soluble drugs and macromolecular biological therapeutics via the topical and transdermal routes (Table 2, entries 13–29) [1,40]. For topical delivery, ILs are usually used as solubilizing and skin-enhancing agents. Biocompatible choline-containing carboxylic acids, such as lactic acid, oleic acid, formic acid, and propionic acid, are used as the internal nonaqueous phase of IL-in-oil (IL/O) microemulsions (MEs) with IL-based surfactant choline oleate to deliver acyclovir (ACV) topically [36]. This formulation significantly enhances the topical delivery of ACV, by 9-fold, compared with water-in-oil MEs. Similarly, choline octanoate IL improves the penetration of navitoclax (a BCL-2 inhibitor) across the skin for an extended period [111]. This formulation has a higher cancer-cell-killing efficacy in topical delivery than in oral delivery. Choline-containing citronellic acid, glutamic acid, caprylic acid, hexenoic acid, glycolic acid, and octanoic acid ILs have been used as solubilizing and skin-enhancing agents of framework nucleic acids (FNAs), resulting in the enhanced penetration of FNAs to the dermis layer with long-term stability [112]. CAGE IL has been used to deliver many macromolecule therapeutics, such as insulin, siRNA, and dextrans [40]. A CAGE IL-based formulation significantly improves the transdermal delivery of dextran with various molecular weights up to 150 kDa, due to the potential use of ILs as an effective and noninvasive transdermal drug delivery system for large hydrophilic molecules (Figure 8A) [113]. CAGE IL-based formulation also enhances the epidermal and dermal penetration of siRNA with suppressed GAPDH expression in mice models compared to the control [114]. A CAGE-containing thrombin-sensitive nanosensor exhibits significant diffusion into the dermis with sustained release into the blood throughout 72 h [44]. This formulation releases reporter molecules into the urine by activating the clotting cascade and retains diagnostic power for 24 h. CAGE IL has also been used for the transdermal delivery of insulin, resulting in enhanced delivery into and across porcine skin compared to CPEs (Transcutol) [115]. This formulation significantly decreased blood glucose levels by 40% within 4 h, with a relatively sustained release of insulin for 12 h, compared to the injection formulation. The transdermal delivery of an antigenic peptide (SIINFEKL) has been formulated using choline fatty acids as biocompatible surfactants. This IL-based system significantly enhanced the skin permeation of the peptide for cancer immunotherapy (Figure 8B) [116]. A recent study replaced the conventional surfactant, Tween-80, with a surface-active bio-IL to develop a thermodynamically stable IL/O ME [117]. The developed IL/O ME generates a ME zone that is two times larger than the Tween-80-based IL/O ME, resulting in 4.7- and 5-fold higher loadings of CLX and ACV, respectively. Another IL/O ME was developed for the transdermal delivery of insulin using choline propionate IL as an internal polar phase and [Cho][Ole] as a surfactant and drug-encapsulating agent [118]. This formulation significantly reduces blood glucose levels, with an improved bioavailability in the systemic circulation and sustained release of insulin for a more extended period, compared to the subcutaneous injection formulation. These results demonstrate that ILs can significantly enhance performance beyond “typical” ME formulations based on traditional surfactants.

### 5.4. Bio-ILs in Vaccine Formulation and Delivery

Vaccination is a therapeutic approach used to stimulate the body’s immune system by delivering an antigen to antigen-presenting cells in order to initiate an immune response. Vaccines can be administered via different routes, such as oral, intramuscular, and transdermal, in different forms, including suspension, nanoparticle, microparticle, and microemulsion. ILs can be used as penetration enhancers, vaccine stabilizers, or adjuvants in different stages of the vaccine formulation (Table 2, entries 30–36) [119,120,121,122]. A novel IL-mediated transcutaneous vaccine formulation, developed using a solid-in-oil nano-dispersion technique in which the model antigen ovalbumin is coated with IL[C_12_MIM][Tf_2_N], triggers the production of a higher level of OVA-specific serum IgG compared to both the PBS control and solid-in-oil (S/O) nano-dispersion without IL [123]. Similarly, a biocompatible choline oleate IL improves the skin permeation of antigenic peptide, by 28-fold, compared to an aqueous vehicle. This IL-based vaccination suppresses tumor growth in vivo compared to 2% wt ethanol containing subcutaneous injection [116]. Recently, choline lactate IL has been used as a safe adjuvant with OVA, resulting in an enhancement of the immune response against the antigen [122]. In another study, choline niacinate IL-based O/IL nanoemulsions were formulated for the intranasal vaccine delivery of influenza split-virus antigens, resulting in high levels of mucosal immune responses with secretory IgA titers 25- and 5.8-fold higher than those of naked and commercial MF59-adjuvanted antigens, respectively [121]. Similarly, humoral immune responses to inactivated foot-and-mouth disease were improved, along with enhanced thermostability and long-term stability compared to the adjuvant of Montanide ISA 206 [120].

**Table 2:** Bio-ILs in various drug formulations and delivery systems.

No.	Drug	Role of ILs	Formulation	Main Findings	Ref.
1.	Favipiravir (Fav)	[Fav]:[Cho], [ProEt], [AlaEt] as pharmaceuticals	Oral	Significantly increased the oral bioavailability up to 1.9-fold compared to the free drug.	[89]
2.	Paclitaxel (PTX)	Choline oleate as an encapsulating agent	Oral	a. Exhibited higher oral bioavailability of PTX than cremophor EL micelles. b. Showed better stability in the intestinal tract.	[102]
3.	Monoclonal antibodies	choline glycolate as a solubilizing agent	Oral	a. Maintained the stability and structure of TNFα antibody with excellent systemic circulation. b. Significantly improved paracellular antibody transport and reduce the viscosity of the intestinal mucus.	[100]
4.	Insulin Immunoglobulin	CAGE as the solubilizing agents	Oral	a. Significantly enhanced the intestinal absorption of macromolecules. b. Improved the diffusion rates in mucin solution by 4-fold compared with control.	[99]
5.	Methotrexate	[MTX]:[ProEt], [TMA], [Cho], [TBP] as pharmaceuticals	Oral	a. ProEt-MTX exhibited 4.6-fold higher oral bioavailability than MTX sodium. b. Reduced systemic toxicity and suppressed tumor growth better than free MTX.	[86]
6.	Sorafenib	CAGE as a solubilizing agent	Oral	a. Enhanced aqueous solubility by 100 times more than the free drug. b. Improved PK profiles in comparison to parent drug suspension. c. Improved drug accumulation in various organs compared to the control.	[97]
7.	Lumefantrine	lumefantrine docusate as pharmaceutical	Oral	a. Enhanced the solubility in lipid-based vehicles up to 80-fold more than the free drug. b. Exhibited improved plasma exposure up to 35-fold compared to control formulations.	[103]
8.	Insulin	CAGE as a solubilizing agent	Oral	a. Improved paracellular transport up to 10-fold more than native insulin. b. Significantly decreased in blood glucose levels (up to 45%) for longer periods (12 h) compared to injection.	[101]
9.	Protein	CAGE as a solubilizing agent	Injection	a. Considered as a safe, non-toxic formulation b. Enhanced insulin absorption by 200% after subcutaneous injections. b. Reduced the interactions of proteins with the subcutaneous collagen.	[107]
10.	Doxorubicin	CAGE as a solubilizing agent	Injection	a. Led to a consistent tumor ablation in a rabbit liver tumor model for a prolong periods. b. Exhibited in a synergistic cytotoxicity with uniform drug distribution in the ablation zone.	[104]
11.	Paclitaxel	[Cho][Gly] as a solubilizing agent	Injection	a. Exhibited improved antitumor activity with a smaller hypersensitivity effect compared to commercial Taxol formulation. b. Exhibited a similar systemic circulation time, slower elimination rate, and antitumor activity to Taxol.	[106]
12.	Doxorubicin	[C_4_MIN][PF_6_] as an agent of the carrier	Injection	a. Exhibited excellent microwave sensitization effects under lower microwave power. b. High inhibition effects in the microwave irradiation.	[105]
13.	Navitoclax (NAVI)	choline octanoate as solubilizing and skin enhancing agent	Topical	a. Exhibited an enhanced skin penetration for an extended period. C. Higher cancer-cell killing efficacy in the topical delivery than oral delivery.	[111]
14.	Thrombin-sensitive nanosensors	CAGE as solubilizing and skin enhancing agent	Topical	a. Provided significant diffusion into the dermis with sustained release into the blood throughout 72 h. b. Released the reporter molecules into the urine by the activation of the clotting cascade.	[44]
15.	Ibuprofen	[Ibu]:[NMP], [Cho] as pharmaceicals	Topical	Exhibited improved skin penetration, and enriched drug accumulation in the target tissue by 2.6 times more than the choline-based drug.	[96]
16.	Framework nucleic acids	[Cho]: carboxylic acid ILs as solubilizing and skin enhancing agents	Topical	a. Exhibited superior structural stability in ILs for a longer period. b. Enhanced FNAs transportation to the lower dermis region in intact, ex vivo porcine skin.	[112]
17.	siRNA	CAGE as solubilizing and skin enhancing agent	Topical	a. Enhanced epidermal and dermal penetration compared with native siRNA. b. Exhibited suppressed GAPDH expression in mice model without toxicity.	[114]
18.	Conotoxins	Matrine oleate	TD	a. High biocompatibility and good fluidity with excellent encapsulation of drug (76%). b. Assisted to penetrate drug across SC barrier and enter the dermis, with a 5.7-fold higher permeability. c. Excellent biocompatibility with improved skin elasticity.	[124]
19.	Levofloxacin	Choline-based poly-IL as drug carrier	TD	a. Exhibited excellent adhesive ability, desirable mechanical properties, and biocompatibility. b. Showed strong antibacterial effect with enhanced transdermal delivery.	[125]
20.	Ovalbumin (OVA)	[EDMPC][Lin] as surfactant and skin enhancing agent	TD	a. Significantly enhanced the transdermal distribution and transdermal flux by 25 and 28-folds, respectively. b. Drastically suppressed tumor growth, and significantly stimulated the OVA-specific tumor immune response in mice model.	[126]
21.	Curcumin	CAGE as solubilizing and skin enhancing agent	TD	a. Significantly enhanced transdermal permeation of curcumin. b. A low percentage of IL was effective in disrupting the skin structure.	[127]
22.	Paclitaxel (PTX)	[Cho][Ole] as surfactant and skin enhancing agent	TD	a. Exhibited a spherical micelles and well-distributed particle size in the range 8.7–25.3 nm. b. Showed 4- and 6-fold higher skin permeation of PTX compared with Tween 80- and ethanol-based formulation, respectively.	[128]
23.	Ovalbumin (OVA)	[Cho][Ole] as surfactant and skin enhancing agent	TD	a. Significantly enhanced the OVA permeation across the skin. b. High level of OVA-specific IgG antibody production with improved tumor growth inhibition compared to the control group.	[129]
24.	Donepezil (DPZ)	dicarboxylic acid-based DPZ as pharmaceutical	TD	Exhibited improved aqueous solubility with excellent skin permeability compared to the free drug.	[78]
25.	Levamisole (Lev)	API-ILs as pharmaceuticals	TD	Oleate-based Lev formulation showed 2.6- and 5.4-fold higher in vitro and in vivo skin permeation capability, respectively, than the Lev salts.	[85]
26.	Gliclazide (Gli)	[Gli][P_6,6,6,14_] as pharmaceutical	TD	a. Exhibited a sustained release profile with a favorable PK profile in the rat compared to oral suspension.	[130]
27.	Ketoprofen, Flurbiprofen Loxoprofen	Triethylamine-APIs as pharmaceuticals	TD	Exhibited enhanced solubility up to 4.50-fold with high skin penetration compared to control patches.	[131]
28.	Insulin	Choline propionate IL as an internal polar phase and [Cho][Ole] as surfactant and drug encapsulating agent	TD	a. Significantly reduced blood glucose levels compared with a commercial surfactant-based formulation. c. Increased the transdermal bioavailability and sustained the insulin level for a much longer period.	[118]
29.	Peptide	[EDMPC]:[Ole], [Lin], [Ste] as surfactants and skin enhancing agents	TD	a. Significantly increased the formulation stability, drug-loading capacity, and encapsulation efficiency. b. Exhibited increased the transdermal delivery flux by 65-fold compared to the control solution.	[132]
30.	Protein	[EDMPC]:[Lin] as surfactants and skin enhancing agents	Vaccine	a. Significantly increased transdermal drug delivery and anticancer immune responses. b. Stimulated a stronger immune response compared with conventional aqueous formulations.	[133]
31.	Influenza split-virus antigen	Choline niacinate as outer phase of the nano emulsion	Vaccine	a. Exhibited improved stability with a reduced and more uniform particle size. b. Showed induced strong mucosal immune responses by 25- and 5.8-fold higher IgA titers 25- than naked and commercial MF59-adjuvanted antigens, respectively.	[121]
32.	Inactivated foot-and-mouth disease virus antigen	Choline niacinate as outer phase of the nano emulsion	Vaccine	a. IL enhanced the thermostability and long-term stability of the antigen. b. Exhibited improved humoral immune responses in mouse models compared to Montanide ISA 206 adjuvant.	[120]
33.	Ovalbumin	Choline lactate as adjuvant	Vaccine	Maintained the stability and structural integrity of the OVA with enhanced the immune response against the antigen.	[122]
34.	Ovalbumin Imiquimod	[Cho][Ole] as surfactant and imiquimod as adjuvant	Vaccine	a. Significantly enhanced the permeation of the antigenic protein and adjuvant across the skin. b. Exhibited high level of OVA-specific IgG antibody production with excellent tumor growth inhibition compared with the control group.	[129]
35.	Peptide	[Cho]: laurate, oleate as surfactants and skin enhancing agents	Vaccine	a. Exhibited enhanced skin permeation by 28-fold compared with aqueous vehicle. b. Suppressed tumor growth in vivo compared to injection.	[116]
36.	Inactivated foot-and- mouth disease virus (iFMDV)	[Cho]: Phosphate, sulfate, and chloride as stabilizing agents	Vaccine	a. Significantly improved the thermo- and long-term storage stability of iFMDV. b. Not affected the immunogenicity of inactivated iFMDV or immune response in vivo.	[134]

Abbreviations: TD, transdermal; [Cho], choline; [ProEt], proline ethyl ester; [TMA], tetramethylammonium; [TBP], tetrabutylphosphonium; CAGE, choline geranate, [Gly], glycine; [NMP], -methyl-2-pyrrolidonium; [Ole], oleate; [Lin], linoleate; [Ste], stearate; [EDMPC], 1,2-dimyristoyl-sn-glycero-3-ethyl-phosphatidylcholine; [P_6,6,6,14_], tributyl(tetradecyl)phosphonium.

## 6. Conclusions and Future Outlooks

The main goal of this review was to emphasize the benefits of ILs over conventional organic solvents/agents in drug formulations and delivery systems. The current issues associated with solid-state pharmaceutical drugs, including limited solubility and permeability, polymorphism, poor bioavailability, and instability, can be addressed by replacing toxic organic solvents and CPEs with ILs or converting APIs into IL forms. However, ILs are inherently toxic and non-biodegradable, which prevents their use in commercial pharmaceutical formulations and clinical applications. Additionally, it has not been established whether studies on ILs employing small animal models would translate to humans. ILs must undergo safety investigations before final formulations are approved for human use. In-depth and extensive studies on the thermodynamics and kinetics of IL-based formulations are required. Future research should focus on developing novel techniques and tools for screening appropriate IL-forming cations and anions to prepare safer ILs or for accurately characterizing and quantifying the impurities of ILs for pharmaceutical applications. Using biocompatible IL-forming compounds of natural origin, or those that have received approval for use in foods or commercial formulations, can help reduce the potential toxicity issues associated with ILs. Recently, renewable plant resource-oriented biocompatible ILs using terpene, betaine, phosphatidylcholine, lecithin, carboxylic acids, choline, fatty acids, and amino acids have been investigated as potential solubilizing and penetration-enhancing solvents or agents, offering a wide range of biocompatible ILs with the potential for biopharmaceutical applications. An in-depth study of IL-based delivery systems is still needed, including oral, parental, pulmonary, ophthalmic, and nasal drug delivery systems. The pharmaceutical use of ILs has concentrated chiefly on transdermal drug delivery. To promote the use of ILs in commercial pharmaceutical applications, clinical studies of IL-based drug delivery systems are required.

## Data Availability

Not applicable.
